# Molecular breeding of barley for quality traits and resilience to climate change

**DOI:** 10.3389/fgene.2022.1039996

**Published:** 2023-01-05

**Authors:** Geng Meng, Søren K. Rasmussen, Cecilie S. L. Christensen, Weiyao Fan, Anna Maria Torp

**Affiliations:** ^1^ Department of Plant and Environmental Sciences, University of Copenhagen, Frederiksberg C, Denmark; ^2^ College of Horticulture, Henan Agricultural University, Zhengzhou, China

**Keywords:** anthocyanin, lignin, grain protein, *Hordeum vulgare*, mutation, seed, food, feed

## Abstract

Barley grains are a rich source of compounds, such as resistant starch, beta-glucans and anthocyanins, that can be explored in order to develop various products to support human health, while lignocellulose in straw can be optimised for feed in husbandry, bioconversion into bioethanol or as a starting material for new compounds. Existing natural variations of these compounds can be used to breed improved cultivars or integrated with a large number of mutant lines. The technical demands can be in opposition depending on barley’s end use as feed or food or as a source of biofuel. For example beta-glucans are beneficial in human diets but can lead to issues in brewing and poultry feed. Barley breeders have taken action to integrate new technologies, such as induced mutations, transgenics, marker-assisted selection, genomic selection, site-directed mutagenesis and lastly machine learning, in order to improve quality traits. Although only a limited number of cultivars with new quality traits have so far reached the market, research has provided valuable knowledge and inspiration for future design and a combination of methodologies to achieve the desired traits. The changes in climate is expected to affect the quality of the harvested grain and it is already a challenge to mitigate the unpredictable seasonal and annual variations in temperature and precipitation under elevated [CO_2_] by breeding. This paper presents the mutants and encoded proteins, with a particular focus on anthocyanins and lignocellulose, that have been identified and characterised in detail and can provide inspiration for continued breeding to achieve desired grain and straw qualities.

## Introduction

Barley (*Hordeum vulgare* L.) is one of the most important agricultural crops grown worldwide and among grain crops currently ranks fifth after maize, wheat, rice and soybean in terms of tonnes produced (FAOstat, 2019). Barley has been adapted so that it can be grown and produce yields in a wide range of climatic zones from the Arctic to Spain, in countries around the Mediterranean, and at high altitudes of 4,200–4,500 m in Peru and Tibet where it tolerates low temperatures and high radiation. Barley covers 64.3% of Tibet’s total crop-growing area and plays an important role in the human diet (Wu, 2010; Shen et al., 2016). It is primarily grown for its starchy grains, which are an important source of energy for livestock, and much less so for human consumption. Barley also provides a significant amount of protein, but due to an unbalanced amino acid composition is of low nutritional value. A significant amount is grown for its malting quality for beer brewing and whiskey production (Newton et al., 2011; Miralles et al., 2021). The release of the malt variety “Alexis” by a small German breeding company Saatzucht Breun in 1986 is a unique example of a quality barley could be successful for decades in most European countries and be the first choice for malsters. In contrast to the world’s other four main crops, barley is rich in dietary fibre. The colours of barley cultivars and landraces are associated with the accumulation of flavonoid pigments in the pericarp and aleurone layer of the seed. Barley grains are a rich source of beta-glucans, which offer numerous benefits to human health globally (Hughes and Grafenauer, 2021). Some cultivars can provide resistant starch to help control obesity and diabetes.

This paper compares the history of molecular barley breeding with a recent review of ten genetic tools and genomic approaches used in the world’s three leading cereal crops of rice, wheat and maize (Benavente and Giménez, 2021) from mutagenesis and transgenesis to QTL mapping, GWAS and genomic selection (Supplementary Table S1). It reveals that to date barley has received the same level of attention as the other main cereal crops in terms of year of first publication within a given approach and number of publications (Supplementary Table S1). Three important mapping methods for quantitative traits were introduced in all cereal crops at around the same time, e.g. 1991 (QTL mapping), 2010 (GWAS) and 2009 (genomic selection/prediction). It should be mentioned that the association genetics method and the forerunner of GWAS, linkage disequilibrium mapping, was explored some years earlier in barley due to this crop’s well distributed chromosomal DNA markers. Expression analysis of cloned genes started around 1983, qRT-PCR in 2004 and RNA-seq in 2013. Application of the search term “mutagenesis” shows that its use began in the 1960s, but with “induced mutation” as the search term, a publication about this method and its use with barley dates back as early as the 1940s (Supplementary Table S1). Genbanks around the world host more than 45,000 barley cultivars, landraces and mutant lines, offering a large genetic resource with which to establish a dedicated breeding nursery. With these available resources, barley has become a model plant, particularly for grain qualities for which there are no better alternatives. Brachypodium with its tiny genome is an attractive model for temperate grasses, however the content of starch, storage protein and lipids (Girin et al., 2014) is very different from that of barley seed.

At the moment, worldwide agriculture is negatively affected by the climate change, which is likely to result in a significant decrease in crop yield or quality characters. Reduced crop production can lead to higher food prices and increased global food insecurity, which, if persistent, will drastically accelerate food prices in future and, in severe cases, social unrest and famine. Global warming, variation in rainfall patterns and the elevated [CO_2_] may largely lead to this disaster. There is a need to enforce enviromics studies (Resende et al., 2022) with multiple environmental information, analyse plant responses to environmental stress on small to large field scale to predict the interactions of genotypes with environments. Barley breeding for climate change will need automated methods and predictors to deal with interactions between genetics and environment (Cooper and Messina, 2021) and such proximal and remote sensors together with computation that makes it feasible to integrate breeding and crop physiology in order to maintain yield stability are being developed for crops. Cammarano et al. (2019) used crop simulation models (CSM) to explore the impacts of climate variability and changes have on barley yield and reported that future barley productivity will decline between 25% and 8% according to climate projections in the Mediterranean basin. Scientists and plant breeders have estimated the severity of climate change on barley yields differently among agro-climatic regions of the world, but unless actions are taken to improve the resilience of the cultivars, climate change will affect the food and feed supply of barley (Yawson et al., 2016; Marcinkowski and Piniewski, 2018; Zhang and Lu, 2022). With its wide range of environmental adaptations, a variety of uses and users, barley has become an attractive model for learning and coping with different climate change impacts (Dawson et al., 2015).

Traditional methods to mitigate climate-related stress, such as growing barley adapted to different regions and seasons, is a way of stress avoidance but this approach can't really deal with the many facets of climate change. Barley quality traits could play a critical role in future breeding, especially under climate change conditions, for example, flavonoids are not only responsible for the colors of different organs, but also contain a variety of specialized metabolites with plentiful biological functions, they especially have powerful roles in stress protection (Winkel-Shirley, 2002). Here we present traits where breeding efforts can take advantage of existing diversity e.g. anthocyanin and lignin contents, protein and starch components to improve cultivar adaptability to various environmental stresses. This review focus on grain quality traits, starting with the identification of mutants and subsequent research aimed at improving those particular traits in barley. We also review some selected genes and traits that are related to particular climate-related stresses, and the diversity of these in the barley gene pool. Prospects for breeding barley with improved grain quality are good in that the demand for health-promoting products and plant-based diets generally remains strong and barley genetic resources will remain a unique resource.

## Anthocyanins in barley grains

Barley grain at maturity may have different pigmentations and colours, including yellow, purple, red and blue, colours that can also be found in the leaf sheath, stem and pericarp of the seed. The yellow pigments are proanthocyanidins synthesised in the seed coat (testa layer) (Aastrup et al., 1984); the purple, red and blue colours are caused by anthocyanins synthesised in the pericarp, glumes and aleurone layer (Harlan, 1914). White barley grains have no pigment. The scientific interest in pigments is driven by their protective functions *in planta* under diverse biotic and abiotic stress conditions, as well as their valuable health benefits (Koes et al., 2005). One of the breeding aims for barley malt is to reduce proanthocyanidins located in the testa layer of the grain, since it can cause haze and degrade the quality of the beer (von Wettstein, 2007).

Flavonoid biosynthesis and the metabolic pathway have been well studied in barley (Jende-Strid, 1993) to describe structural genes encoding enzymes of the pathway, as well as regulatory genes governing spatial and temporal patterns of the structural gene expressions. Anthocyanins are flavonoid pigments conferring different colours on plant tissues. They provide a well-known physiological function to attract pollinators and seed dispersers (Chaves-Silva et al., 2018). Apart from their colorant properties, anthocyanins are reported to be induced under diverse biotic and abiotic stresses, including UV radiation, cold and drought stress, and bacterial and fungal pathogen infection, which suggests a role in cell stress-coping mechanisms (Sperdouli and Moustakas, 2012; Sivankalyani et al., 2016; Bæksted Holme et al., 2021; Lin et al., 2021). Moreover, anthocyanins also play an important role in human health to prevent different chronic pathologies (Li et al., 2021). Specifically for barley, anthocyanin synthesis can be affected by various environmental factors, including light, temperature and nutrients. Light is an essential factor in anthocyanin synthesis in the leaf blade and sheath, while low temperatures promote anthocyanin synthesis in the blade and exogenous gibberellic acid increases anthocyanin synthesis mainly in the sheath (Martínez and Favret, 1990). In Tibet, more than 68% of wild barley varieties are colored (Choo, 2002), and this region is well-known for its harsh environmental conditions, including low temperature, UV-B radiation and high-altitude. Over the altitude of 4,000 m, all barley grains are dark-colored (Choo, 2002). This indicates that the coloration of barley grain may be the result of plants’ response to environmental adaptation.

Mutations in structural and regulatory genes related to flavonoid biosynthetic pathways can readily be induced in barley. Since 1977, more than 700 mutants associated with this pathway have been isolated, including mutants without anthocyanin and proanthocyanin, which were designated as *ant* mutants. These mutants with recessive mutations at the “Ant” loci were named *Ant*1 to *Ant*30 (Jende-Strid, 1998). Mutations in ten of the *Ant* genes have resulted in the synthesis of proanthocyanidins being blocked in the testa layer of the grains, whereas mutations in 18 of the *Ant* genes reduce anthocyanin synthesis in different organs of the plants (Jende-Strid, 1990).

The first such mutant (*ant*13-13) was identified in 1974 and blocked the synthesis of anthocyanins, catechins and proanthocyanidins (Jende-Strid, 1978). Jende-Strid (1993) reports that *Ant*17, *Ant*18, *Ant*19, *Ant*22, and *Ant*26 are structural genes, while *Ant*13 is a regulatory gene that regulates the transcription of more than three structural genes in the flavonoid pathway (Jende-Strid, 1993). The *Ant*1 gene, which codes for a R2R3 MYB-type transcription factor, has been mapped to barley chromosome 7HS and activates transcription of four structural genes in leaf sheaths (Himi & Taketa, 2015; Shoeva et al., 2015). The purple colour of the grain pericarp depends on the *Ant*2 gene, which is another regulatory gene encoding a basic Helix-Loop-Helix (bHLH) transcription factor (Cockram et al., 2010). In the flavonoid pathway, MYBs are able to interact with bHLHs and WD40 repeat proteins to form highly dynamic MYB/bHLH/WD40 (MBW) complexes. The *HvAnt2* gene, together with the *HvMpc1*/*HvAnt1* gene, activates the transcription of structural genes (Himi & Taketa, 2015; Shoeva et al., 2015). One of the few examples of marker assisted selection in barley is the combination of alleles of the *Ant*1 and *Ant*2 loci (Gordeeva et al., 2019) showing that antocyanin only accumulate in the pericarp when dominant alleles of both genes are present. Zhou et al. (2021) reported that *Ant*1 interacts with *Ant*2, and overexpression of *Ant*1 activates the transcription of *Ant*2, resulting in an increased anthocyanin accumulation in the pericarp and aleurone layer of transgenic barley grains. Proanthocyanidins are not synthesised in the grains of *ant*28 lines, and the *HvAnt28* gene has been identified as another MYB-type factor (Himi et al., 2012). While the *Ant1* and *Ant2* genes determine the accumulation of anthocyanins in the pericarp, the *Ant28* gene controls the biosynthesis of proanthocyanidins (condensed tannins) in the barley seed coat. HvMpc2 (MYB), HvMyc2 (bHLH) and HvWD40 form the MBW complex, which is responsible for anthocyanin biosynthesis in the aleurone in barley. These are necessary for regulating the expression of structural anthocyanin biosynthesis genes, therefore it should be possible to obtain anthocyanin-enriched barley grains by manipulating the expression of the regulatory gene.

*Ant*17, *Ant*18 and *Ant*30 have been found to be structural genes. *Ant*17 codes for a flavanone-3-hydroxylase (F3H) subunit, and the mutant allele is a mutation in the structural domain of the gene (Meldgaard, 1992). *Ant18* encodes dihydroflavonol-4-reductase (DFR) and complements the anthocyanin-free mutation when delivered into leaf sheath tissue by microprojectile bombardment, as evidenced by the synthesis of anthocyanin in bombarded tissues (Wang et al., 1993). The barley *ant30* phenotype is caused by mutations in the chalcone isomerase gene (Druka et al., 2003).

Proanthocyanidin and anthocyanin are synthesised by the flavonoid pathway, and it is generally accepted that anthocyanidin formation may occur if the proanthocyanidin is blocked because the intermediate is diverted by oxidation to form anthocyanidins (Jacques et al., 1977). However, genetic prevention of anthocyanin synthesis apparently does not automatically lead to accumulation of proanthocyanidin as McClure (1975) reports on a mutant blocked in both proanthocyanidin and anthocyanidin synthesis. It would be of interest to isolate and study a mutant that is blocked in proanthocyanidin synthesis but not in anthocyanin synthesis.

Combinations of classical and molecular methods have been used to generate new varieties with enhanced anthocyanin accumulation as well as different colours in barley. The transgenic approach can severely limit product marketing due to consumer perception and the difficulties of marketing genetically-modified foods. In recent years, CRISPR/Cas9 has been regarded as a potential method to generate non-transgenic plants. Gasparis et al. (2018) demonstrate the effectiveness of an optimised RNA-guided Cas9 system that can be used to generate homozygous knockout mutants in the progeny of transgenic barley plants. AtMYBL2 and FaMYB1 act as transcriptional repressors and negatively regulate the biosynthesis of anthocyanin in Arabidopsis and strawberry (Aharoni et al., 2001; Matsui et al., 2008). Knockdown of this kind of repressor of anthocyanin biosynthesis could boost the anthocyanin content. Altogether, a better understanding of the mechanisms underlying the anthocyanin repression will facilitate breeding in barley using non-transgenic methods.

For all the above reasons, increased anthocyanin content is a significant goal in barley breeding and biotechnology. A better understanding of the genetic regulation of anthocyanin biosynthesis is likely to become one of the most interesting targets for future breeding work to breed food for high nutrient density in cereals, potentially with a transformative impact on the health of consumers. The flavonoid biosynthesis pathway is a branch of the phenylpropanoid pathway, which is the precursor for numerous specialised metabolites, including flavonoids, tannins, coumarins and monolignols (Vogt, 2010; Gray et al., 2012). The shared enzymatic steps and split of the pathway to anthocyanin and lignin, which is an important trait in straw, has recently been discussed (see Meng et al. (2021) and references therein).

## Barley straw quality

Straw from grain production is often considered a simple by-product of the harvested crop, but it is used in bioindustries to produce biofuel, textile, paper and biochemicals (for a detailed list see Anwar et al. (2014); Guerriero et al. (2016)). Improving the stiffness of barley culm to reduce lodging due to an increased harvest index, strong winds and rainfall may take advantage of the large number of known mutations, as reviewed in the study of Dockter and Hansson (2015) in which, however, stem lignin is not considered. Lignocellulose is the main component of plant cell walls and the most abundant organic material on earth. It is primarily composed of energy-rich polysaccharides in the form of cellulose, hemicellulose and pectin, large rigid structures of lignin and structural (glyco) proteins crosslinked with phenolic compounds. Lignocellulose is an essential source of animal feed and is used in various bioindustries (Guerriero et al., 2016). The structure of lignin makes it recalcitrant to bioconversion and it is the major limitation in utilising nutritional polysaccharides for animal feedstock and the production of bioproducts. Transcriptomic analysis revealed that chilling of tobacco plants could induce lignin biosynthesis (Zhou et al., 2019) and in barley, Janská et al. (2011) reported that the expression profiles of genes participating in lignin synthesis were changed significantly under cold treatment. For those reasons, lignin biosynthesis has attracted considerable attention, making it one of the most studied pathways.

## Mutants in lignin

Naturally-occurring mutants with reduced lignin were identified in cereals such as barley and maize in the early 20^th^ century (Buckley, 1930; Jorgenson, 1931; Emerson et al., 1935). Barley (*Hordeum vulgare* L.) mutants linked to reduced lignin exhibit an orange coloration in the internode, lemma, palea and rachis, and therefore the locus is called “Orange lemma one” and the locus symbol *rob1*. Even though *rob1* mutants have been known for almost a century, only a few studies have investigated its utility with regard to animal feed or biofuel production (Meyer et al., 2006; Daly et al., 2007; Stephens and Halpin, 2008). Lignin content has been found to be 10%–15% lower in *rob1* mutants of different backgrounds and the *rob1* mutation is mapped to the *HvCAD2* gene (Stephens and Halpin, 2008), similar to *bm1* in maize.

Monolignols are synthesised from phenylalanine or tyrosine (exclusively for grasses) through the general phenylpropanoid pathway, as mentioned above, followed by transport to, deposition and polymerisation in the cell wall. The steps involved in the synthesis are well documented (Boerjan et al., 2003; Vanholme et al., 2010; Wang et al., 2018; Xie et al., 2018).

Biochemical and genetic investigations of the monolignol pathway/phenylpropanoid pathway/lignin/straw quality have identified and characterised several genes in the pathway by expression analysis and biochemical assays. Genes in the monolignol pathway have been regulated in order to decrease lignin or alter the composition, making the pathway a perfect target for precise genome editing that can build on existing studies (Vanholme et al., 2008). Promising target genes for reduction of lignin recalcitrance are the final genes in the pathway encoding cinnamyl alcohol dehydrogenase (CAD) and the caffeic acid O-methyltransferase (COMT) (Fu et al., 2011a; Fu et al., 2011b). CAD is responsible for reducing cinnamaldehydes to cinnamyl alcohols, the precursors of the building blocks of lignin, also called monolignols, whereas COMT is a multifunctional enzyme, but with a preference for methylations of 5-hydroxyconiferaldehyde to sinapaldehydes, and therefore primarily affects the synthesis of syringyl monolignol (Mottiar et al., 2016). However, CAD is part of a small gene family, making it more complex to engineer, and the number of genes varies between species, with only two genes in maize and 11 in barley (Bukh et al., 2012). COMT was thought to be a single copy gene, but is actually a low-copy-number gene in some species (Wu., 2010; van Parijs et al., 2015).

The economic advantages of increasing the nutritional value of cereals and replacing fossil fuels with biofuels have encouraged scientists to investigate and regulate nine of the genes in the monolignol biosynthetic pathway, making it an extensively studied pathway. Lignin genes in maize (8) and switchgrass (7) have mostly been studied in a transgenic approach, with only a few studied in rice (4) and barley (1), as reviewed by Christensen and Rasmussen (2019). Generally, downregulating or knocking out genes leads to a reduced lignin content. RNAi is the primary method to downregulate the expression of genes. However, repression of gene expression does not give a complete picture of a gene’s function. Instead, using CRISPR/Cas9 to directly knock out a gene by creating a stable mutation is a more advantageous way of studying a gene function (Belhaj et al., 2015). In contrast to chemically-induced mutations, site-directed mutagenesis by CRISPR/Cas9 requires knowledge of the candidate gene’s DNA sequence and is capable of making a precise point mutation, with stable inheritance over a few generations. Besides targeting coding sequences it allows for extending the allelic diversity by including promotor sequences. Breeding for climate change resilience most likely involves quantitative traits and the strength of expression of particular genes. This would revive the strong relation between research in the lab and translating knowledge into breeding. The breeder would bridge between molecular research over greenhouse proof-of-concept to selection in the field. CRISPR/Cas9 is predicted to revolutionise precision breeding, but it has been stated that this type of mutation is not excluded from the scope of the EU’s GMO regulation (Chamber, 2018), which still complicates the use of this technology outside academia. Instead, it is suggested that existing germplasms be screened using TILLING to identify new mutations and overcome current regulatory difficulties around crop improvements.

## Storage protein of barley

High temperature and water deficient can remarkably change the grain quality by changing the composition and concentration of starch and protein. The grain protein content of modern barley cultivars typically varies between 8% and 14%, depending on the rate of fertilisation. Half of the protein is in storage proteins known as hordeins, as reviewed by Gubatz and Shewry (2010). Ahmed et al. (2013) reported that the concentration of hordein significantly increased under drought alone and under drought and salt stresses in all of the tested genotypes of Tibetan wild and cultivated barleys. Since there is less than 1% lysine in the protein, the nutritional value of barley protein is low. It therefore became a breeding goal to improve barley grain protein for livestock feed, identifying high-lysine barley mutants (Doll 1973). However, a pleiotropic effect of these mutations was a yield penalty that could not be offset by breeding efforts (Munck, 1992). Screening for recombination events within the loci carrying large gene families was unsuccessful. With molecular biology tools emerging in the late 1970s, the idea was to clone the hordein genes with an inferior nutritional value and introduce codon mutations to increase the lysine content. However, it became clear that each of the hordein loci carries a large number of genes, making achievement of this strategy an overwhelming task. With the arrival of CRISPR-Cas9 site-directed mutagenesis, the large-scale introduction of mutation to increase lysine codons in gene families may be feasible.

## Barley food

There is increased interest in using barley for food due to its content of beta-glucan, resistant starch, antioxidants and vitamin E, for example. Studies have shown that replacing some of the wheat flour with barley flour can indeed improve the nutritional quality of bread and biscuits, for example, and that although the addition of barley flour affects the properties of dough and bread as well as the sensory quality, the final products are generally still acceptable to consumers (Do et al., 2016; Narwal et al., 2017; Liu et al., 2020; Yu et al., 2021). Commercially, barley is already used in a range of products. A recent survey sampling 2462 products from the four main supermarkets in metropolitan Sydney showed that 13.4% of breakfast cereals, 14.3% of breads and 0.8%–3.0% of savoury biscuits, grain-based muesli bars, flour and plant-based milk alternatives contained whole grain barley, while this ingredient was absent from the sampled noodles/pasta (Hughes and Grafenauer, 2021). In addition, BARLEYmax®, a whole grain barley line high in beta-glucan and resistant starch developed by CSIRO, was present in a few samples (0.4%–2.4%) of breakfast cereals, bread and grain-based muesli bars. On the global market (Europe, United States, Brazil, Canada, Indonesia, Malaysia, New Zealand and Singapore), 2.6% of products from similar food categories found in the Mintel Global New Product Database contained whole grain barley, while only a few contained BARLEYmax® (Hughes and Grafenauer, 2021).

Wild barley germplasm also known as crop wild relatives may provide lots of useful genes for barley improvement the mitigate the challenges of different environmental conditions. Zhao et al. (2010) identified two Tibetan wild barley genotypes, XZ5 and XZ16, that were highly tolerant to drought and salinity. Subsequently, Ahmed et al. (2013) revealed that both Tibetan wild barley showed a higher total antioxidant capacity and higher contents of starch, protein, total phenol, amylases and amino acids compared to other barley cultivars. In addition, the elevated CO_2_ concentration result in a significant decline of the content of grain protein resulting in decreased malting and brewing quality. Therefore, the traits related to barley food quality are not only important for human being health, but also play important roles in stress response. Another example of introgression of wild barley *Hordeum chilense* into durum wheat and thereby creating tritordeum with new grain characteristics for food product development (Ávila et al., 2021).

Mineral malnutrition is a serious problem affecting a large section of the population, particularly in developing countries. Iron deficiency anaemia has been estimated to affect 18.1% of children under 5 years old and 19.2% of pregnant women on a global scale (Black et al., 2013), while 17.3% of the population is estimated to be at risk of inadequate zinc intake based on a study of zinc availability in food supplies in a given region (Wessells and Brown, 2012). One way to overcome this is to exploit natural variation for grain mineral content/concentration. Gyawali et al. (2017) found grain Zn content to range from 10.4 to 54.5 mg/kg and Fe content from 21.9 to 91.0 mg/kg in a collection of 336 cultivated spring barley germplasms, while Mamo et al. (2014) found an even greater variation (Zn: 6.23–71.96 mg/kg; Fe: 27.2–109.60 mg/kg) in grains from a collection of 298 Ethiopian and Eritrean landraces. Using GWAS, one QTL for Zn was found on 6H (Mamo et al., 2014), while Gyawali et al. (2017) identified three QTLs for Zn on 2H, 3H and 5H, as well as 11 QTLs for Fe on 1H, 2H, 4H, 6H and 7H in their GWAS population. Combined with information from a couple of previous studies in which biparental mapping populations were used to identify QTL for grain zinc (Lonergan et al., 2009; Sadeghzadeh et al., 2015), these results may be utilised for marker-assisted breeding for grain zinc and Fe content in future. Ways of improving micronutrient concentration in staple crops has been reviewed in detail by Graham and Welch (2004) and explored in HarvestPlus (www.ifpri.org).

Biofortication by breeding is complicated by the fact that barley also accumulates antinutritional factors, such as phytic acid, that severely reduces the bioavailability of Fe and Zn (Brune et al., 1992). Mutants in seed phytic acid have been identified in barley (Rasmussen and Hatzack, 1999; Dorsch et al., 2003), however, as with the hordein mutants above, these low-phytic acid mutants may also suffer from pleiotropic effects on grain yield. Since yield has always been the primary breeding target, improving quality traits that may reduce yield is problematic. The above-mentioned breeding strategies are presented as a way of solving some of the technical problems of using barley grain, however breeding for these traits can easily take eight to 10 years to come to market. In parallel, industrial enzymes to solve the same problems have been developed and can be used with any barley cultivar. Industrial beta-glucanase can be added to barley to overcome the problem of haze in brewing and to barley feed to improve poultry growth. Furthermore, microbial phytase can be added to feed and food to hydrolyse phytic acid and thereby remove the antinutritional factor, thus making the bound phosphate available for uptake.

As is the case for wheat and rye, foods containing barley should be strictly avoided by patients with coeliac disease as gluten triggers an abnormal immune response in these patients, resulting in a range of symptoms (Rubin and Crowe, 2020). In barley, immunodominant epitopes are found in C-hordeins, but both β- and γ-hordeins also contain epitopes (Tye-Din et al., 2010). In a recent study, Tanner et al. (2016) developed a new ultra-low gluten (ULG) barley line by intercrossing three individual mutants lacking β-hordeins, C-hordeins and D-hordeins respectively. The new line (ULG 3.0) showed extremely low levels of hordeins and shrunken seed phenotype. However, repeated crossing with wild-type barley improved the seed size to an extent where it may in future be used to produce food and beverages for patients with coeliac disease (Tanner et al., 2016).

## Conclusion and future directions

**FIGURE 1:**
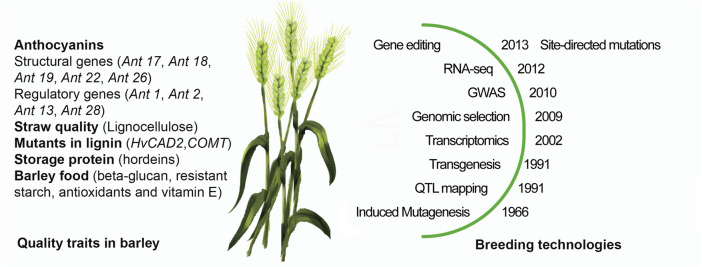
Breeding for grain and straw qualities in Barley. Three targets for grain and two for straw are highlighted together with relevant barley mutations to the left of the barley plant and to the right the year of the first publications of key breeding technologies from induced mutagenesis to the recent site-directed mutagenesis in barley.

As summarized in Figure 1 barley has been suitable for all new breeding technologies that have been introduced for crop plant improvement for decades. Barley has been adapted to a wide range of agro-climatic conditions and will yield a wide range of compounds that is useful for humans and animals and serve industrial purposes. When dealing with climate change, breeders have to cope with two types of conditions imposed on the barley production: an annual steady and predictable increase in [CO2], and at the same time unpredictable variations in temperature, precipitation and storms. Temperature show variation from year to year as well as during the growth season, where as an example temperature hikes during flowering have strong negative impact on the yield. The site-directed mutation technology used on regulatory sequences allows to create a series allele with different strength in expression to mitigate these climate effects. Continued breeding must be done to achieve desired grain and straw qualities and the following short list of topics seems particular important and timely taken current policies and available technologies into consideration:

• Grain Protein Content
• Nutritional qualities for health products
• Side-streams of lignin polymer

There is again in Europe a strong desire to increase plant protein production and at the same time control nitrogen fertilizers in agriculture to minimize losses to the environment. New technologies for precision agriculture make it worth pursuing improvements by combining genetics, nitrogen use efficiency (NUE) and in field spectral analysis Grieco et al. (2022) to optimize on these simultaneously to obtain significant increase in grain protein content and yield (Sandhu et al., 2021). In recent years NUE candidate genes has been identified in barley collections (Wang et al., 2012; Pauli et al., 2014; Han et al., 2016) but the dissection and use in breeding probably requires use of artificial intelligence. Crop simulations models for enviromic-aided genomic prediction are under intense development (Costa-Neto et al., 2021). Although previous attempt to also improve amino acid composition did not reach commercial varieties, maybe site-directed mutagenesis could offer large scale mutagenesis in the storage protein gene-family. Heath products rich in flavonoids or resistant starch is also demanded by consumer world-wide. Many of the genes controlling these compounds are known for barley and improvement can be done by using natural variations combined with site-directed mutagenesis in order to produce better resources for tailor made health products. The focus on straw lignin has been to reduce the recalcitrance to bioconversion either to make it more digestible for cattle or to cut down into monomers that can be fermented into bioethanol. However, lignin as such is also a valuable polymer that could provide interesting monomers for building new materials. Rather than reducing lignin content of the straw, increasing the lignin content would make barley straw a valuable side-stream for developing new industrial products.
