# Supplementary material for: Molecular breeding of barley for quality traits and resilience to climate change
Source: Front Genet. 2023 Jan 5;13:1039996. doi: 10.3389/fgene.2022.1039996 (PMC9851277; doi:10.3389/fgene.2022.1039996)
Supplement: Supplementary file 1 [file Table1.DOCX]

**Supplementary Table S1.** Year of first publication and citation for barley (this study). Year of first publication for rice, wheat and maize (Benavente and Gimenez, 2021).

| **Approach** | **Rice** | **Wheat** | **Maize** | **Barley** |
| --- | --- | --- | --- | --- |
| QTL mapping | 1990 | 1992 | 1987 | 1991 |
| GWAS | 2009 | 2009 | 2011 | 2010 |
| Genomics selection | 2014 | 2011 | 2007 | 2009 |
|  |  |  |  |  |
| Gene expression | 1984 | (1972) | (1971) | 1983 |
| Transcriptomics | 2001 | 2002 | 2003 | 2002 |
| Quantitative PCR | 2003 | 2003 | 1999 | 2004 |
| RNA-seq | 2010 | 2011 | 2011 | 2012 |
|  |  |  |  |  |
| Mutagenesis | 1971 | 1964 | 1961 | 1966 |
| Transgenesis | 1988 | 1990 | 1988 | 1991 |
| Gene editing | 2012 | 2013 | 2014 | 2013 |
|  |  |  |  |  |
| Lignin | 1968 | 1931 | 1927 | 1935 |
